# Toxicity of Certain Penta-Coordinated Organotin(IV) and Tetra-Coordinated Tin(II) Complexes of Heterocyclic β-Diketones Against the Larvae of *Aedes Aegypti* (Liston)

**DOI:** 10.1155/MBD.1999.183

**Published:** 1999

**Authors:** Asha Jain, Sanjiv Saxena, Audhesh K. Rai, Prabhu N. Saxena, J. Venkateshwara Rao

**Affiliations:** 1 Department of Chemistry University of Rajasthan Jaipur 302 004 India; 2 Toxicology laboratory Department of Zoology Institute of Basic Science Agra University Agra 282 004 India; 3 Indian Institute of Chemical Technology (IICT) Hyderabad India

## Abstract

Some organotin(IV) and tin(II) complexes of composition R_3_Sn[R'COC:CON(C_6_H_5_)N:CCH_3_] (where R=C_4_H_9_, R'=CH_3_, C_3_H_5_, p-ClC_6_H_4_; R=C_6_H_5_, R'=C_6_H_5_ and p-ClC_6_H_4_) and Sn[p-ClC_6_H_4_COC:CON(C_6_H_5_)N:CCH_3_]_2_ were screened for their toxicity against *Aedes aegypti larvae*. Organotin(IV) complexes were more active than tin(II) complexes.


### TOXICITY OF CERTAIN PENTA-COORDINATED ORGANOTIN(IV) AND TETRA-COORDINATED TIN(II) COMPLEXES OF HETEROCYCLIC β-DIKETONES AGAINST THE LARVAE OF *AEDES AEGYPTI* (LISTON)

Asha Jain[1], Sanjiv Saxena[1], Audhesh K. Rai*[1], Prabhu N. Saxena[2] and J. Venkateshwara Rao[3]

[1] Department of Chemistry, University of Rajasthan, Jaipur-302 004, India
[2] Toxicology laboratory, Department of Zoology, Institute of Basic Science, Agra University, Agra-282 004, India
[3] Indian Institute of Chemical Technology (IICT), Hyderabad, India

Abstract
Some organotin(IV) and tin(II) complexes of composition $R_3Sn[R'CO\underline{C:CON(C_6H_5)N:C}CH_3]$ (where R = $C_4H_9$, R' = $CH_3$, $C_3H_5$, p-$ClC_6H_4$; R = $C_6H_5$, R' = $C_6H_5$ and p-$ClC_6H_4$) and Sn[p-$ClC_6H_4CO\underline{C:CON(C_6H_5)N:C}CH_3]_2$ were screened for their toxicity against *Aedes aegypti larvae*. Organotin(IV) complexes were more active than tin(II) complexes.

### Introduction

Although the chemistry of organotin(IV) complexes with various organic ligands[1-3] has experienced a very large growth, the literature cited on the corresponding tin (II)[4,5] complexes is less abundant. The tin complexes have received significant attention mainly due to their structural[6-8] and stereochemical[9-10] considerations as well as due to their potential biological applications. A number of reports have been cited in the literature concerning the biological activities of organotin(IV) complexes of amino acids[11], carboxylic acids[12] and oxinates[13]. Organotin complexes also exhibit antitumor[14-16], fungicidal[17,18] and insecticidal[19,20] activities. The present paper focuses attention on the structure-activity relationship of organotin(IV) and tin (II) complexes of sterically demanding heterocyclic (-diketones with the larvae of *Aedes aegypti* (liston) which is an important mosquito species and is responsible for the transmission of dengue fever in man in the tropical and subtropical world, including India.

Materials and Methods

The organotin(IV) and tin(II) complexes of compositions $R_3Sn[R'CO\underline{C:CON(C_6H_5)N:C}CH_3]$ where R =- $C_4H_9$, R' = p-$ClC_6H_4$ (compound I); R = $C_4H_9$, R' = $C_6H_5$ (compound II); R = $C_4H_9$, R'= $CH_3$ (compound III); R = $C_6H_5$, R' = p-$ClC_6H_4$ (compound IV); R = $C_6H_5$, R' = $C_6H_5$ (compound V) and Sn[p-$ClC_6H_4CO\underline{C:CON(C_6H_5)N:C}CH_3]_2$ (compound VI) were synthesised[4-6] and the structures of these complexes have also been reported by us earlier[4,6]. The ligand (LH) employed for the preparation of these complexes was prepared by a reported[21] procedure where LH = $RCO\underline{C:C(OH)N(C_6H_5)N:C}CH_3$, R = $CH_3$ ($L_1H$); $C_6H_5$ ($L_2H$) and p-$ClC_6H_4$ ($L_3H$).

The toxicity of these complexes was assessed against *Aedes aegypti larvae (liston)*. For this purpose, filter paper strips containing eggs of this mosquito were obtained from the vector control research centre Pondicherry, India. They were moistened with water and the newly emerged larvae were transferred to rearing trays supplied with an adequate quantity of yeast powder. The colony was maintained in mosquito cages at a temperature of 28±1°C and relative humidity 80-85%.

The adults were fed on water soaked resins and 10% glucose solution soaked in cotton pads. The females were offered back and belly shaved rabbits for blood meals during alternate days. Ovipositional bowls containing water and lined with blotting paper strips were kept daily in mosquito cages for collection of eggs. They were removed each morning and kept for larval emergence. Light was provided to the colony by two 60 watt fluorescent tubes and a 60 watt bulb with a photoperiodicity of 10-10.5 hours of darkness, 1-1.5 hours of dawn, 10-10.75 hours of light and 1-1.25 hours of darkness.

The Aedes larvae were kept in beakers in the laboratory for experimentation and for each ppm concentration 10 larvae were put in each beaker. Replicates and controls were run simultaneously.

The compounds were dissolved in acetone and ppm solutions were prepared. The larvae were exposed to 10,20,30 to 150 ppm concentrate solutions for 24 hours. The mortality was counted in each beaker. The data was subjected to statistical tests and the toxicity of the complex was assessed by log probit method[22].

### Results and Discussion

The ligands $L_1H$, $L_2H$ and $L_3H$ were found to be inactive when tested against *Aedes aegypti Larvae (liston).* The results of the present investigation are listed in Table 1 and the compounds are arranged in descending order of activity.

**Table 1**

| **No.** | **Compounds** | **$LC_{50}$ Value (in ppm)** |
|---|---|---|
| I | $Bu_3Sn[p\text{-}ClC_6H_4COC{:}CON(C_6H_5)N{:}CCH_3]$ | 54 |
| II | $Bu_3Sn[C_6H_5COC{:}CON(C_6H_5)N{:}CCH_3]$ | 60 |
| III | $Bu_3Sn[CH_3COC{:}CON(C_6H_5)N{:}CCH_3]$ | 60 |
| IV | $Ph_3Sn[p\text{-}ClC_6H_4COC{:}CON(C_6H_5)N{:}CCH_3]$ | 80 |
| V | $Ph_3Sn[C_6H_5COC{:}CON(C_6H_5)N{:}CCH_3]$ | 110 |
| VI | $Sn[p\text{-}ClC_6H_4COC{:}CON(C_6H_5)N{:}CCH_3]_2$ | 145 |

While discussing structure-activity relationships of the tin complexes, the following factors were taken into consideration (Fig.1):

A The presence or absence of the organic group R on the tin atom.
B The nature of the organic group R appended to tin.
C The coordination number and geometry of the tin complexes.
D The effect of substitution on the ligand i.e. nature of R'.
E The presence or absence of halogen atom in the complex.

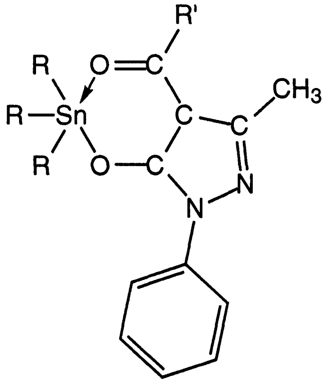

$R = C_4H_9$, $R' = p\text{-}ClC_6H_4$ (Compound I);
$R = C_4H_9$, $R' = C_6H_5$ (Compound II);
$R = C_4H_9$, $R' = CH_3$ (Compound III);
$R = C_6H_5$, $R' = p\text{-}ClC_6H_4$ (Compound IV);
$R = C_6H_5$, $R' = C_6H_5$ (Compound V);

**Fig.1:** (4-acyl-2,4-dihydro-5-methyl-2-phenyl-3H-pyrazol-3-onato) triorganotin(IV) compounds

These complexes possess a five coordinate tin centre as inferred from $^{119}Sn$ NMR spectral studies[6] of complexes I and III. In these complexes, three butyl groups are attached to the central tin atom. The most plausible explanation for the activity of these complexes I, II and III may be the presence of organotin moiety, $(C_4H_9)_3Sn$. This moiety is considered to be an important contributor to activity. Further, among the tributyl tin complexes, some substitutions were carried out on the ligand. Compound I was more active than II and III. Compound I possesses a chlorine atom ($R' = p\text{-}ClC_6H_4$) whereas compound II ($R' = C_6H_5$) and compound III ($R' = CH_3$) do not possess chlorine atoms. It is evident from this observation that the presence of a chlorine atom plays some role in the toxicity of the compounds[20].

Compounds containing aryl groups are usually less toxic than corresponding ones with lower alkyl groups (i.e. $C_1$ -$C_4$). In order to study this general toxicological trend, complexes IV and V were prepared and studied. These complexes were found to be less active than compounds I, II and III. This observation is consistent with the general trend. In these complexes, three phenyl groups are bonded to the tin atom. They are, however, structurally similar to tributyl tin complexes in having a five coordinated tin centre.

Further, complex IV is more active than V. The only difference between these two complexes is the presence of chlorine in the former on the ligand moiety.

Complex VI (Fig.2) was the least active. This complex does not possess any organic group (R) on the central tin atom. This observation clearly reveals that the presence of organic groups on the tin atom is an important factor which imparts enhanced activity to the organotin complex.

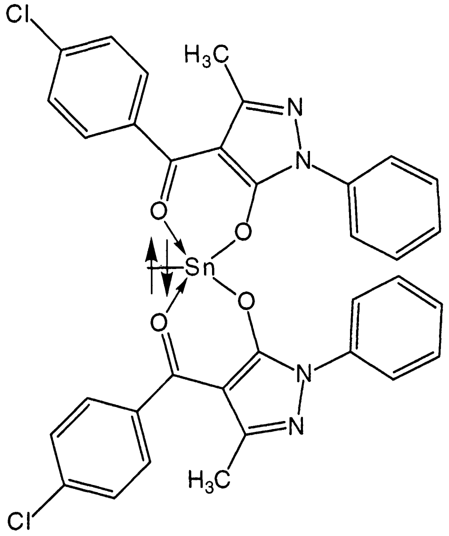

**Fig.2** bis(4-p-chlorophenyl-2,4-dihydro-5-methyl-2-phenyl-3H-pyrazol-3-onato)tin(II)

In the tin(II) complex, the central tin atom is surrounded by four oxygen atoms. The noteworthy feature of this complex is of course the presence of a lone pair of electrons on the central tin atom. Although this complex may exist in trigonal bipyramidal or square pyramidal geometry. The spectral evidences are not conclusive[4] as regards the existence of these geometrical forms. However, crystallographic study of the analogous tin complex, bis(4-acetyl-2,4-dihydro-5-methyl-2-phenyl-3H-pyrazol-3-onato)tin(II) reveals that it possesses a distorted trigonal bipyramidal geometry at tin with the lone pain of electrons occupying an equatorial site[23].

On the basis of the above discussion, it may be inferred that the organotin(IV) complexes are more active than a tin(II) complex derived from the same organic ligands. Tributyl groups attached to the central tin atom contribute more to activity than triphenyl groups. Complexes having a chlorine atom on the ligand moiety have a slightly enhanced activity over complexes lacking such atom. The tin complex in the divalent state possesses a lone pair of electrons and was the least active. However, the general implications of a lone pair on the biological activity of such complexes need further detailed study.

### Acknowledgement

One of the authors, Asha Jain is thankful to the CSIR, New Delhi for financial assistance.

### References

1. G.K. Sandhu and N.S. Boparoy, *J. Organomet. Chem.*, **423** (1992) 183.
2. T.S.B. Baul, D. Dey, D.D. Mishra, *Synth. React. Inorg. Met.- Org. Chem.*, **23** (1993) 53.
3. A. Saxena, S. Saxena and A.K. Rai, *Indian J. Chem.*, **31** (1992) 469.
4. A. Jain, S. Saxena and A.K. Rai, *Main Group Met. Chem.*, **14** (1991) 329.
5. A. Jain, S. Saxena and A.K. Rai, *Indian J. Chem.*, **32A** (1993) 439.
6. A. Jain, S. Saxena and A.K. Rai, *Indian J. Chem.*, **30A** (1991) 881.
7. A. Jain, S. Saxena and A.K. Rai, *Main Group Met. Chem.*, **16** (1993) 223.
8. S. Bhambhani, S. Saxena and A.K. Rai, *Main Group Met. Chem.*, **18** (1995) 251.
9. S. Saxena, R. Bohra and A.K. Rai, *Inorg. Chim. Acta*, **173** (1990) 191.
10. A. Jain, S. Saxena, R. Bohra and A.K. Rai, *Main Group Met. Chem.*, **18** (1995) 139.
11. F. Huber, G. Roge, L. Carl, G. Atassi, F. Spreafico, S. Filippeschi, R. Barbieri, A. Silvestri, E. Rivarola, G. Ruisi, F. Di Bianca and G. Alonzo, *J. Chem. Soc. Dalton Trans.* (1985) 523.
12. C. Dep, A. Adhikari and B.Basu, *Main Group Met. Chem.*, **18** (1995) 135.
13. M. Gielen, R. Willem, J. Holecek and A. Lycka, *Main Group Met. Chem.*, **16** (1993) 29.
14. M. Gielen, M. Acheddad, B. Mahiev and R. Willem, *Main Group Met. Chem.*, **14** (1991) 73.
15. M. Gielen, R. Willem, *Appl. Organomet. Chem.*, **7** (1993) 201.

16. M. Gielen, A. Bouhdid, F. Kayser, M. Biesemans, D. de Vos, B. Mohieu and R. Willem, *Appl. Organomet. Chem.*, **9** (1995) 251.
17. L. Moens, H. Maraite, B. Mahieu, A. El Khloufi, R. Willem and M. Gielen, *Main Group Met. Chem.*, **15** (1992) 275.
18. A. Chakrabarti, Sk. Kamruddin, T.K. Chattopadhyaya, A. Roy, B.N. Chakarborty K.C. Molloy and E.R.T. Tiekink, *Appl. Organomet. Chem.*, **9** (1995) 357.
19. P.N. Saxena and A.J. Crowe, *Appl. Organomet. Chem.*, **2** (1988) 185.
20. P.N. Saxena and S. Saxena, *Appl. Organomet. Chem.*, **3** (1989) 279.
21. B.S. Jensen, *Acta Chem. Scand.*, **13** (1959) 1668.
22. J.J. Finney, Probit Analysis, Cambridge University press, (1971) 303.
23. A. Jain, S. Saxena, R. Bohra and A.K. Rai, *Main Group Met. Chem.*, **18** (1995) 661.

**Received: August 24, 1998 - Accepted: September 14, 1998 - Received in revised camera-ready format: May 25, 1999**

